# A new genome assembly of an African weakly electric fish (*Campylomormyrus compressirostris*, Mormyridae) indicates rapid gene family evolution in Osteoglossomorpha

**DOI:** 10.1186/s12864-023-09196-6

**Published:** 2023-03-20

**Authors:** Feng Cheng, Alice B. Dennis, Josephine Ijeoma Osuoha, Julia Canitz, Frank Kirschbaum, Ralph Tiedemann

**Affiliations:** 1 Unit of Evolutionary Biology and Systematic Zoology, Institute of Biochemistry and Biology, University of Potsdam, Potsdam, Germany; 2 Laboratory of Adaptive Evolution and Genomics, Research Unit of Environmental and Evolutionary Biology, Institute of Life, Earth & Environnment, University of Namur, Namur, Belgium; 3 Senckenberg German Entomological Institute, Müncheberg, Germany; 4 Department of Crop and Animal Science, Faculty of Life Sciences, Humboldt University, Berlin, Germany

**Keywords:** *Campylomormyrus*, Pacbio sequencing, Gene family, Osteoglossomorpha, *Kv1*

## Abstract

**Background:**

Teleost fishes comprise more than half of the vertebrate species. Within teleosts, most phylogenies consider the split between Osteoglossomorpha and Euteleosteomorpha/Otomorpha as basal, preceded only by the derivation of the most primitive group of teleosts, the Elopomorpha. While Osteoglossomorpha are generally species poor, the taxon contains the African weakly electric fish (Mormyroidei), which have radiated into numerous species. Within the mormyrids, the genus *Campylomormyrus* is mostly endemic to the Congo Basin. *Campylomormyrus* serves as a model to understand mechanisms of adaptive radiation and ecological speciation, especially with regard to its highly diverse species-specific electric organ discharges (EOD). Currently, there are few well-annotated genomes available for electric fish in general and mormyrids in particular. Our study aims at producing a high-quality genome assembly and to use this to examine genome evolution in relation to other teleosts. This will facilitate further understanding of the evolution of the osteoglossomorpha fish in general and of electric fish in particular.

**Results:**

A high-quality weakly electric fish (*C. compressirostris*) genome was produced from a single individual with a genome size of 862 Mb, consisting of 1,497 contigs with an N50 of 1,399 kb and a GC-content of 43.69%. Gene predictions identified 34,492 protein-coding genes, which is a higher number than in the two other available Osteoglossomorpha genomes of *Paramormyrops kingsleyae* and *Scleropages formosus*. A Computational Analysis of gene Family Evolution (CAFE5) comparing 33 teleost fish genomes suggests an overall faster gene family turnover rate in Osteoglossomorpha than in Otomorpha and Euteleosteomorpha. Moreover, the ratios of expanded/contracted gene family numbers in Osteoglossomorpha are significantly higher than in the other two taxa, except for species that had undergone an additional genome duplication (*Cyprinus carpio* and *Oncorhynchus mykiss*)*.* As potassium channel proteins are hypothesized to play a key role in EOD diversity among species, we put a special focus on them, and manually curated 16 *Kv1* genes. We identified a tandem duplication in the *KCNA7a* gene in the genome of *C. compressirostris*.

**Conclusions:**

We present the fourth genome of an electric fish and the third well-annotated genome for Osteoglossomorpha, enabling us to compare gene family evolution among major teleost lineages. Osteoglossomorpha appear to exhibit rapid gene family evolution, with more gene family expansions than contractions. The curated *Kv1* gene family showed seven gene clusters, which is more than in other analyzed fish genomes outside Osteoglossomorpha. The *KCNA7a*, encoding for a potassium channel central for EOD production and modulation, is tandemly duplicated which may related to the diverse EOD observed among *Campylomormyrus* species.

**Supplementary Information:**

The online version contains supplementary material available at 10.1186/s12864-023-09196-6.

## Background

Teleost fishes comprise more than half of the vertebrate species in the world, showing a marvelous biodiversity concerning morphology, ecology and behavior [1]. It has been shown that a teleost-specific whole genome duplication (TS-WGD) had occurred in the common ancestor of all extant teleost [2–5]. Although there is no solid evidence to support the connection between the TS-WGD and the successful radiation of teleosts, the former provided enormous opportunities for gene innovation and evolution [6]. The redundant duplicated genes may be free to evolve new or related functions in the course of long-term modification and divergence, and may hence have fostered functional and phenotypic diversification in teleost fish [7].

One of the possible trajectories of diversification following gene duplication is parallel evolution among disparate taxa [8], as exemplified in the evolution of electric organs in unrelated lineages [9]. Among fish, myogenic electric organs have independently evolved at least six times, enabling the generation of electric fields, which are used for communication, navigation, and in extreme cases for predation and defense [10–14]. This electric organ-specific parallel evolution appeared both in elasmobranch fish and two unrelated teleost lineages: the Gymnotiformes from South America and the Mormyroidei from Africa [10].

The vast majority of African weakly electric fishes belongs to the Mormyridae, one of the most diverse family of freshwater fishes. They are endemic to Africa where there are at least 188 described species [15]. The genus *Campylomormyrus* comprises 15 described species, most endemic to the Congo Basin [15, 16]. As in other mormyrids, the electric organ of *Campylomormyrus* is derived from myogenic tissue and located in the caudal peduncle [17]. It is composed of specialized electrocytes which produce externally measurable electric organ discharges (EODs) [17]. The species-specific EOD displays a huge diversity in signal duration and waveform [18, 19]. However, the proximate mechanisms underlying the divergence of EOD among species are only partially understood [20–23]. In order to better understand the evolution of this genus, a high-quality genome is imperative. Up to now, three complete genomes have been published of electric fishes of the genera *Paramormyrops*, *Electrophorus* and *Brachyhypopomus* [24–26]. Hence, our genomic knowledge is still too incomplete for a comprehensive assessment of electric fish’s molecular evolution and its impact on phenotypic divergence.

**Fig. 1:**
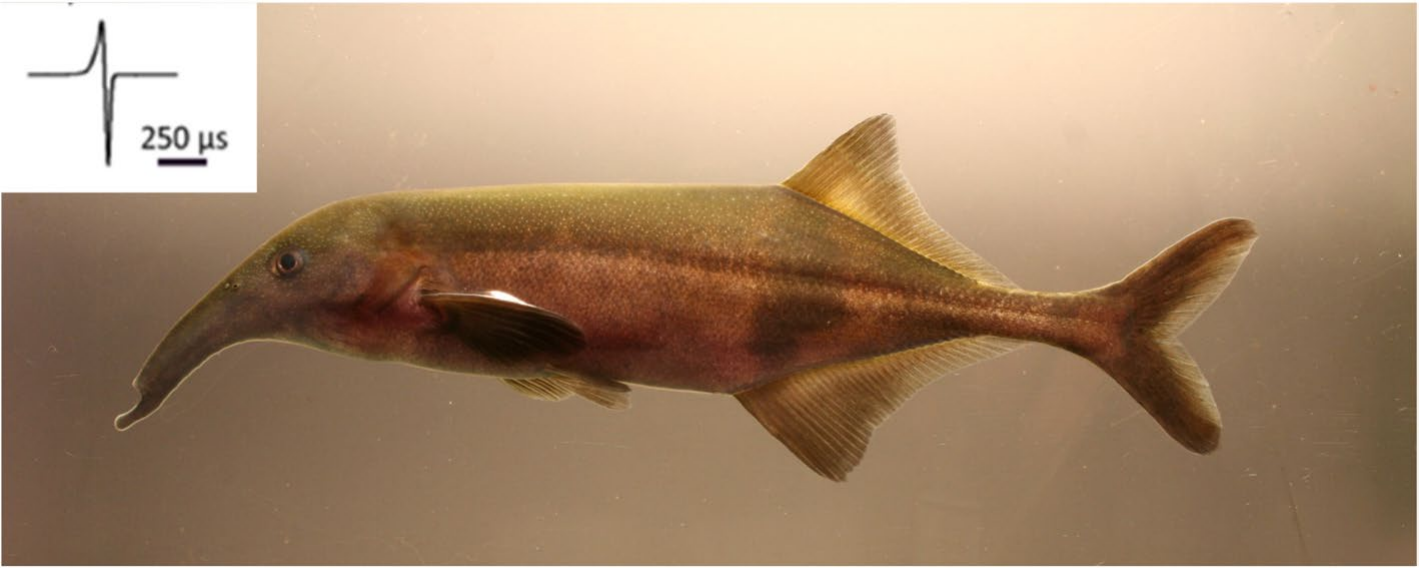
Photo and typical electric organ discharge (EOD, upper left corner) of the sequenced species *Campylomormyrus compressirostris.* (Photo taken by Frank Kirschbaum)

The aim of our study is to generate a high-quality genome for the African weakly electric fish species *Campylomormyrus compressirostris*, a species that produces a biphasic pulse type EOD (Fig. 1). This genome will provide an invaluable resource for the genus *Campylomormyrus*, an established model for adaptive radiation and ecological speciation [27]. As a first step, we here use this genome to compare the evolution of gene family size in *C. compressirostris* relative to other electric fishes, and to teleost fish in general. In addition, we have manually curated and examined the important *Kv1* voltage-gated potassium channel genes, which is hypothesized to be involved in the diversification of the EOD signal and speciation in weakly electric fish [19].

## Results

### Genome assembly of *Campylomormyrus compressirostris*

Here we report a new genome assembly from the African weakly electric fish *C. compressirostris*. The specimen used for sequencing was artificially bred and raised at the University of Potsdam, Germany. A total of 294 Gb Pacbio raw data (~ 294.3 billion reads) was generated. Circular consensus sequencing (CCS) produced 15.5 Gb (~ 1.03 million reads) high fidelity (HiFi) raw data. The produced HiFi data were analyzed based on their k-mer distribution [28] to estimate the genome size (799 Mb) and genome heterozygosity (0.96%).

Using the hifiasm assembler [29], the final assembly is 862 Mb in size and contains 1,497 contigs with a contig N50 of 1.3 Mb and a GC-content of 43.69% (Table 1). The largest contig has a length of 1,399 kb. The assembly also produced the set of alternate contigs (815.8 Mb). The genome quality was close to that of the *Brachyhypopomus occidentalis* genome, and significantly improved compared with the two other published electric fish genomes: *Electrophorus electricus* and *Paramormyrops kingsleyae* (Table 1).

**Table 1:** Comparison of available genome assemblies for 4 electric fish (Osteoglossomorpha: *C. compressirostris, P. kingsleyae*; Gymnotiformes: *E. electricus*, *B. occidentalis*) and 1 non-electric osteoglossomorph fish (*S. formosus*)

	Osteoglossomorpha			Gymnotiformes	
	*C. compressirostris*	*P. kingsleyae*	*S. formosus*	*E. electricus*	*B. occidentalis*
Sequencing Platform	Pacbio HiFi	Illumina HiSeq2000	Illumina HiSeq2000	Illumina HiSeq2000	10 x
Genome size (Mb)	862	880	779	720	540.3
Coverage	14x	83x	137.6x	55x	46x
Complete BUSCOs	94.6%	95.0%	-	97.0%	93.8%
n contigs	1,497	4,496	-	340,589	1,435
Contig N50 (kb)	1,399	37.6	30.73	104	5,400
GC content	43.9%	43.0%	-	42.5%	44.6%
Genes Predicted	34,492	27,677	22,016	22,000	34,347

The integrity of the assembly was demonstrated by 94.6% Benchmarking Universal Single-Copy Orthologs (BUSCO) [30] completeness, indicating the high degree of completeness of the gene regions.

### Genome annotation

Genome annotation was conducted in several steps. First, repeats were identified and masked. The repeat content was identified based on the RepeatModeler [31] and accounted in total for 27.28% (235.37 Mb) of the assembled genome. Next, gene predictions were made using combined evidence from empirical transcriptomic data and protein references from the National Center for Biotechnology Information (NCBI). These were provided to the MAKER pipeline, which predicted 34,492 protein-coding genes, 280,886 exons and 246,394 introns (Table 2). The coding sequence (CDS) covers 5.3% of the genome. Over 90% of the genes have an annotation edit distance (AED) of 0.5 or lower (Additional file 1), suggesting that they are well supported by either protein or RNA-seq evidence. The number of predicted protein-coding genes is notably higher than in the other two sequenced Osteoglossiformes fishes: *P. kingsleyae* and *Scleropages formosus* which had 27,677 and 22,016 protein-coding genes, respectively (Table 1).

**Table 2:** Genome annotation statistics

	Exons	Introns	Genes	CDS
Number	280,886	246,394	34,492	34,492
Longest in kb	26.4	292.8	424	534
Mean length	225	1,095	9,645	1,330
% genome covered by			38.6	5.3

### Orthogroup identification in teleost fish

The gene family analyzer CAFE5 (Computational Analysis of gene Family Evolution) [32] was used to compare annotated gene content in our genome with 33 genomes of teleost fish that we selected based on contiguity and taxonomic representation (Additional file 2).

Orthogroups (OGs) were clustered among the filtered peptides sequences in OrthoFinder [33]. We obtained 23,613 OGs from teleost fish by OrthoFinder, of which 402 were identified as single copy. There are 500 unique OGs in *C. compressirostris*, and 919 in Mormyridae (represented by *C. compressirostris* and *P. kingsleyae*). A total of 1,169 unique OGs were identified among Osteoglossomorpha, 1,134 among Otomorpha, and 2,540 among Euteleosteomorpha.

### Gene and gene family expansion and contraction analysis

We estimated both expansions and contractions in gene family size across the evolutionary history of all teleosts. All the 23,613 OGs from OrthoFinder were used as input in CAFE5. CAFE5 estimated the gene family turnover rate lambda for each group of Osteoglossomorpha, Otomorpha and Euteleosteomorpha.

Based on the gene family clustering results in CAFE5, 368 OGs were significantly changed in gene numbers per family among teleosts, of which 276 were annotated using the UniProt database (Table 3). From this set, OGs were repeatedly (over 5 times) associated with zinc finger protein, transposon, immunoglobulin and GTPase. We put the relative frequency of OGs with these functions into perspective of their occurrence among the 20,663 annotated OGs in total, employing Fisher’s exact tests [34]. Among the OGs with significantly changed gene number contents across teleost lineages, OGs related to transposons, immunoglobulins and GTPases are significantly overrepresented. In the CAFE5 gene family analysis, the estimated gene family turnover rate lambda was larger for Osteoglossomorpha (0.0029) than for Otomorpha (0.0022) and Euteleosteomorpha (0.0022).

**Table 3:** Number of orthogroups (OGs) with significant changes in gene number (*p* < 0.05) among teleosts, compared to all OGs in teleost fish. Overrepresentation of certain functions was tested with Fisher's exact test

	OGs with significantly changed gene number	All OGs	*P*-value
Total number	368	23,613	
Number with annotation	276	20,663	
Number associated with zinc finger proteins	12	809	0.6416
Number associated with transposons	10	36	< 0.001
Number associated with immunoglobulins	10	90	< 0.001
Number associated with GTPases	7	202	0.0228

In order to eliminate the bias introduced by species that have undergone an additional and more recent WGD, we repeated the CAFE5 analysis without those species (i.e., excluding *Cyprinus carpio* and *Oncorhynchus mykiss*). With this removal, Osteoglossomorpha (0.0030) show an even larger lambda, relative to Otomorpha (0.0020) and Euteleosteomorpha (0.0019), indicating a higher gene family turnover (birth–death) rate in Osteoglossomorpha.

**Fig. 2:**
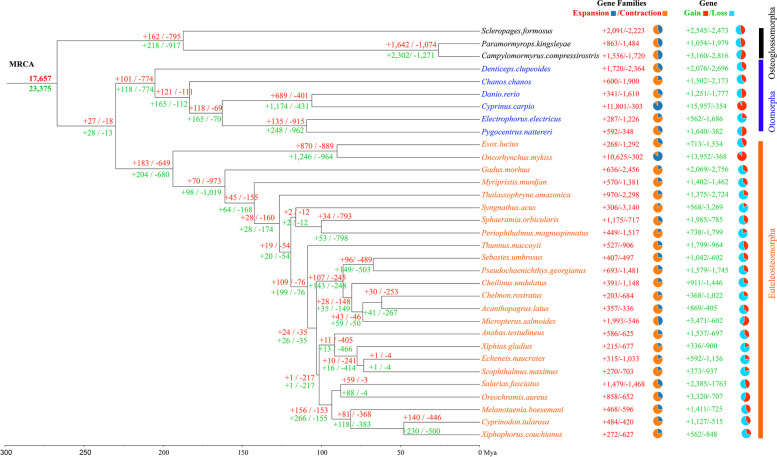
Inferred MRCA (most recent common ancestor) numbers of genes (green) and gene families (red) as well as the expansions (gains, +) and contractions (losses, -) in genomes of different teleost lineages. The pie charts show the gene/gene family expansions and contractions of species compared to the MRCA

We compared the inferred gene and gene family change for each node, relative to its preceding node in the phylogeny. One thousand five hundred fifty-six gene families were expanded in *C. compressirostris* whereas 1,720 contracted (Fig. 2). This species gained 3,160 genes and lost 2,816 genes. The common ancestor of *C. compressirostris* and *P. kingsleyae* had 1,642 gene families expanded and 1,074 contracted, *P. kingsleyae* only had inferred expansion in 863 gene families, relative to contraction in 1,484 gene families (Fig. 2).

**Fig. 3:**
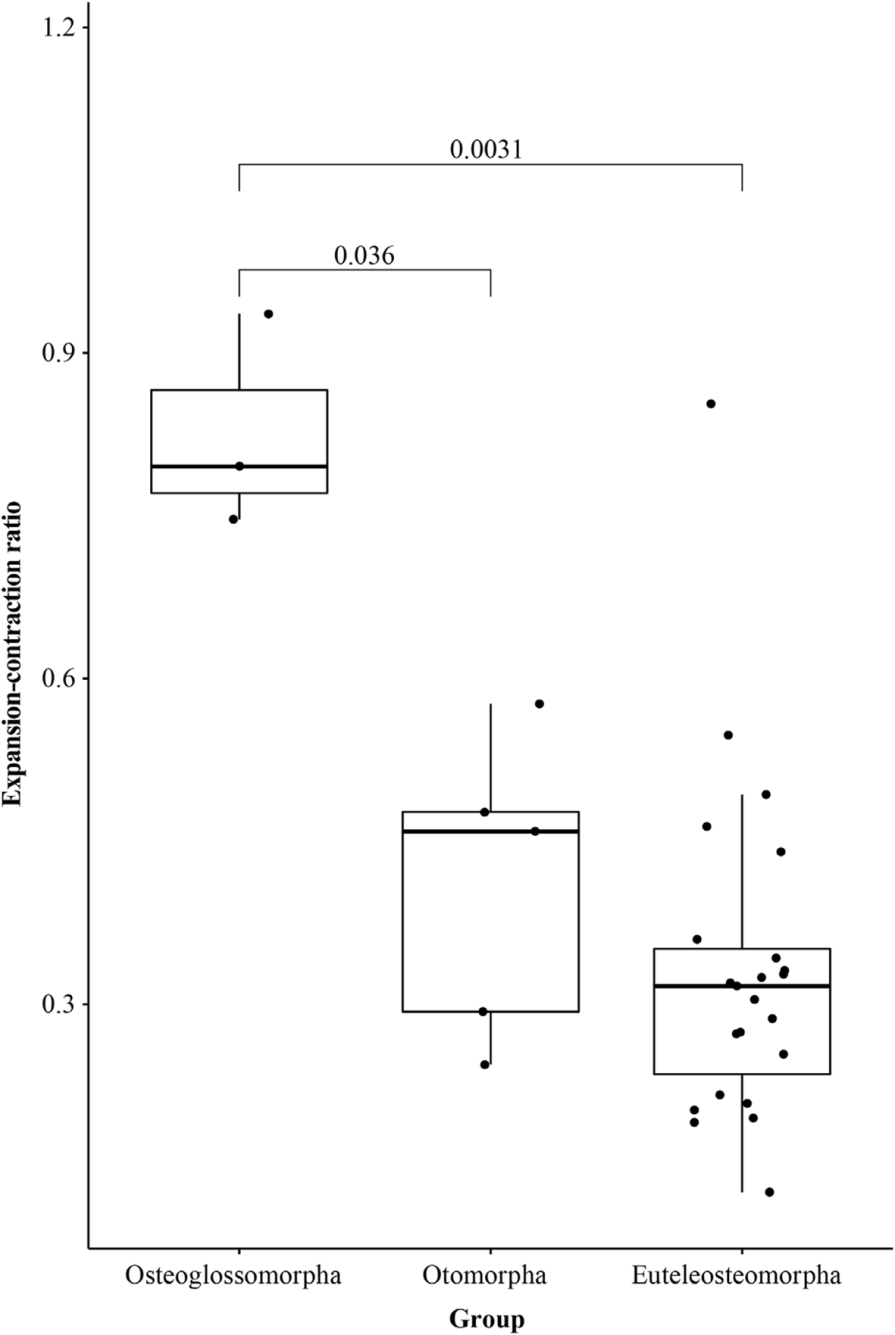
Box-and-scatter plot of gene family expansion/contraction ratios in the groups Osteoglossomorpha, Otomorpha and Euteleostemorpha. The values above box plot are the P-values between corresponding groups from a t-test

For comparing the lineage-specific gene family and gene change relative to their most recent common ancestor (MRCA) of all selected teleost fishes, we summarized the gene and gene family change for every species (Fig. 2, Additional file 3) and counted the ratios of expanded/contracted gene family (as well as gained/lost gene) numbers. In most of the analyzed species, more gene families contracted than expanded, with the exception of the two species with additional, recent genome duplications: *C. carpio* and *O. mykiss*. Leaving these two species out, the ratio of gene family expansions/contractions was significantly higher in Osteoglossomorpha than in Otomorpha and Euteleosteomorpha (*p*-values of 0.036 and 0.0031 respectively, t-Test [35], Fig. 3).

### Gene families and pathways with increased turnover in electric fish and Osteoglossomorpha

**Fig. 4:**
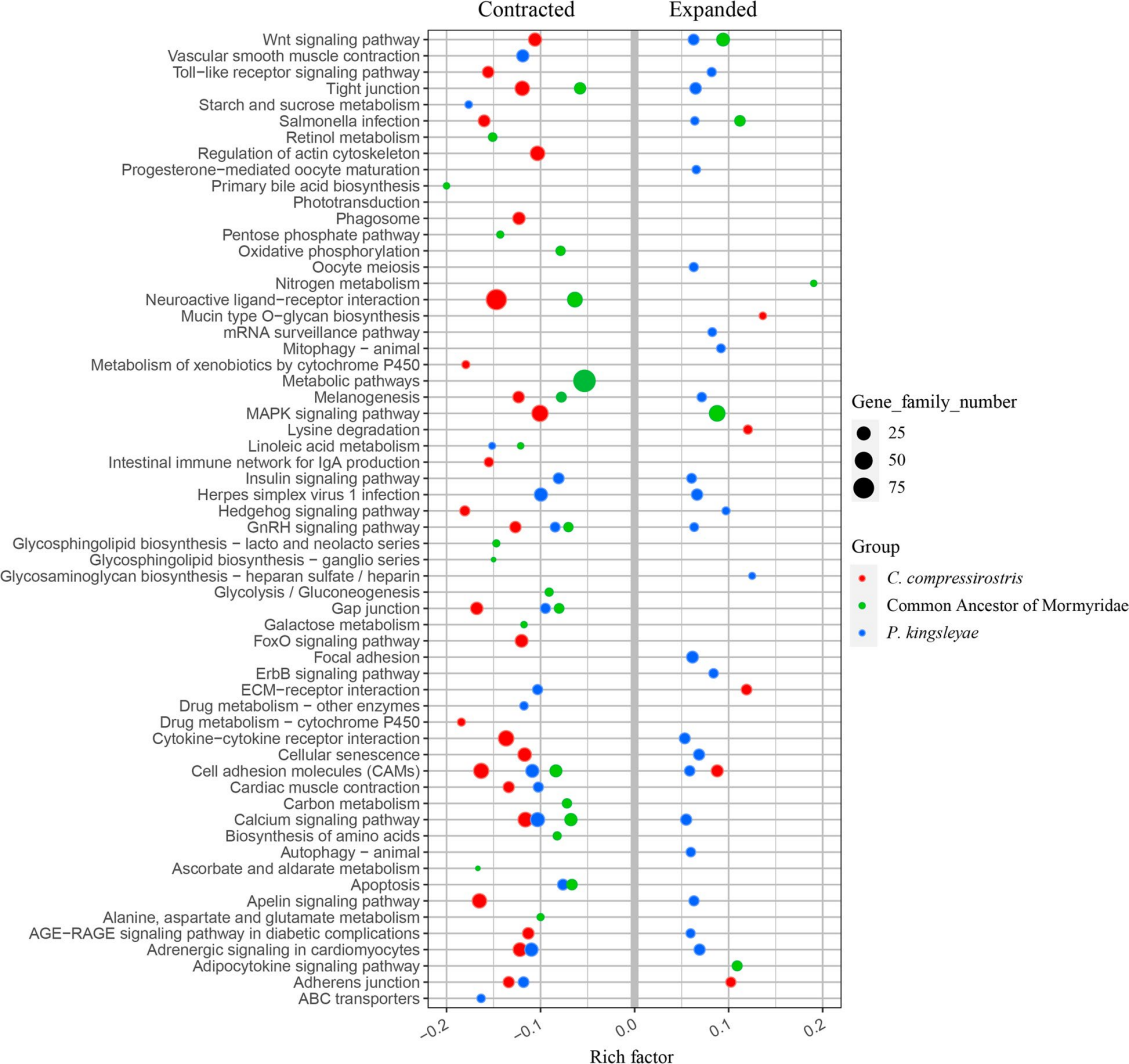
Numbers of expanded and contracted gene families in KEGG pathways with significantly elevated turnover (contraction or expansion) among *C. compressirostris* (red), *P. kingsleyae* (blue) and their common ancestor (green). The plot size represents the gene family number in the respective species. Note that non-significant values are not plotted, hence not all pathways have dots for all taxonomic groups

We assigned gene families to Kyoto Encyclopedia of Genes and Genomes (KEGG) [36] pathways to identify those pathways exhibiting a significantly (*p* < 0.05) elevated turnover (i.e., a significantly higher number of either contracted and expanded gene families) in the two mormyrids *C. compressirostris* and *P. kingsleyae,* relative to their common ancestor. This analysis yielded 60 significantly enriched pathways (Fig. 4), of which 25 contained contracted OGs and 5 expanded OGs in *C. compressirostris*; for *P. kingsleyae,* 16 pathways comprised contracted OGs and 24 expanded ones. For the ancestor node of both mormyrid fishes, 22 pathways with elevated turnover exhibit contracted OGs and 5 expanded ones. The rich factor indicates the degree of the enrichment in the respective KEGG pathways (Fig. 4). The pathway with highest rich factor is primary bile acid biosynthesis in contracted OGs and nitrogen metabolism in expanded OGs.

**Fig. 5:**
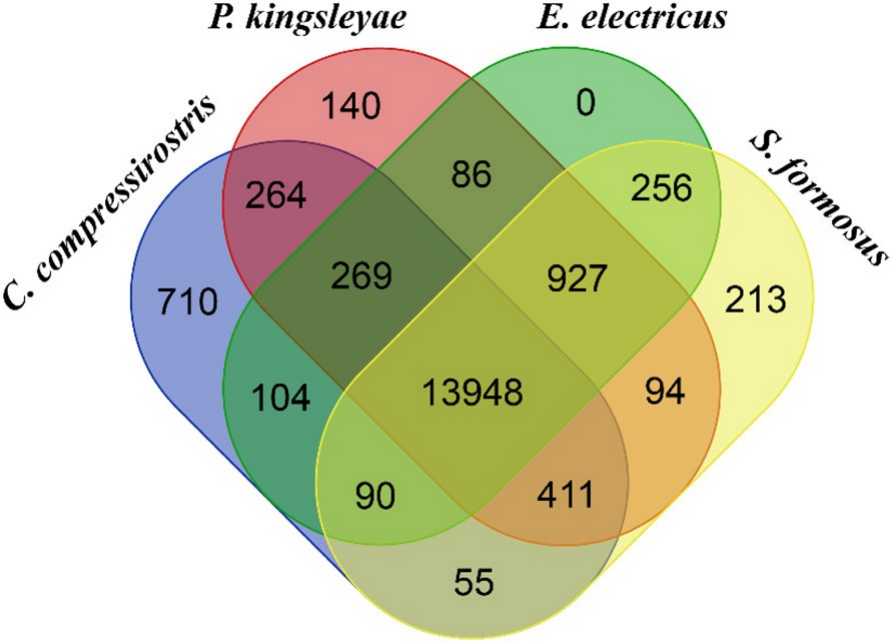
Venn Diagram graph of all orthologous gene families shared/not shared among four species (*C. compressirostris, P. kingsleyae, E. electricus, S. formosus*)

To examine shared OGs, a VennDiagram [37] was created to visualize all OGs in three electric fish (*C. compressirostris, P. kingsleyae* and *E. electricus*) and one non-electric fish (*S. formosus*) from Osteoglossomorpha (Fig. 5). There were 269 enriched OGs shared among the electric fishes and 411 enriched OGs shared among Osteoglossomorpha. 264 enriched OGs were only shared among mormyrids. Although this is a small dataset, it could suggest that OGs turnover patterns are more similar among phylogenetically related groups (here, osteoglossomorphs) than among species having convergently evolved an active electric sense.

### *KCNA* gene cluster curation

The potassium voltage-gated channel subfamily A (*KCNA, Kv1*) encodes shaker-related voltage-gated potassium channels, which are considered as a component of electric organ discharges. 16 complete *Kv1* genes, which contained both start and stop codons, were manually curated in the *C. compressirostris* genome (Table 4, Additional file 4). 11 of them were predicted in the annotation pipeline. Manual searches identified *KCNA3a/b, KCNA7b* and *KCNA10a/b*. We could not find *KCNA5a* gene, which was considered to be lost according to the available resources.

**Table 4:** *KCNA* genes information in the *C. compressirostris* genome

Gene	Contig	Start position	End position	Number of exons	Length of cds
*KCNA1a*	ptg000343l	158,599	160,074	1	1,476
*KCNA1b*	ptg000633l	255,2072	2,553,541	1	1,470
*KCNA2a*	ptg000962l	2,034,837	2,036,324	1	1,488
*KCNA2b*	ptg000135l	6,635,624	6,637,102	1	1,479
*KCNA3a*	ptg000962l	2,057,829	2,059,386	1	1,557
*KCNA3b*	Ptg000135l	6,613,381	6,614,937	1	1,557
*KCNA4a*	ptg000643l	775,778	777,787	1	2,010
*KCNA4b*	ptg000333l	510,057	512,045	1	1,989
*KCNA5b*	ptg000633l	2,507,887	2,509,569	1	1,683
*KCNA6a*	ptg000633l	2,583,269	2,584,375	1	1,107
*KCNA6b*	ptg000225l	702,207	703,643	1	1,437
*KCNA7a_1*	ptg000028l	8,120,428	8,129,001	2	1,539
*KCNA7a_2*	ptg000028l	8,089,350	8,094,209	2	1,542
*KCNA7b*	ptg000600l	1,376,337	1,378,893	2	1,551
*KCNA10a*	ptg000962l	2,004,772	2,006,448	1	1,677
*KCNA10b*	ptg000135l	6,670,889	6,672,565	1	1,677

The CDS length among *KCNA* genes varied from ~ 1,400 bp to ~ 2,000 bp. We detected two *KCNA7a* gene copies in contig ptg000028l with a regional distance of ~ 26 kb (Table 4). Genes *KCNA1a/b, KCNA2a/b, KCNA3a/b, KCNA4a/b, KCNA5b, KCNA6a/b* and *KCNA10a/b* have only one exon, whereas *KCNA7a_1, KCNA7a_2* and *KCNA7b* were found to have two exons. Among the newly discovered duplications of *KCNA7a*, the exon1 of *KCNA7a_1* and *KCNA7a_2* are identical, however, there are 55 single nucleotide polymorphisms (SNPs) among them in the 855 bp of exon2. The p-distance was 0.0657 between two *KCNA7a* copies of exon2.

**Fig. 6:**
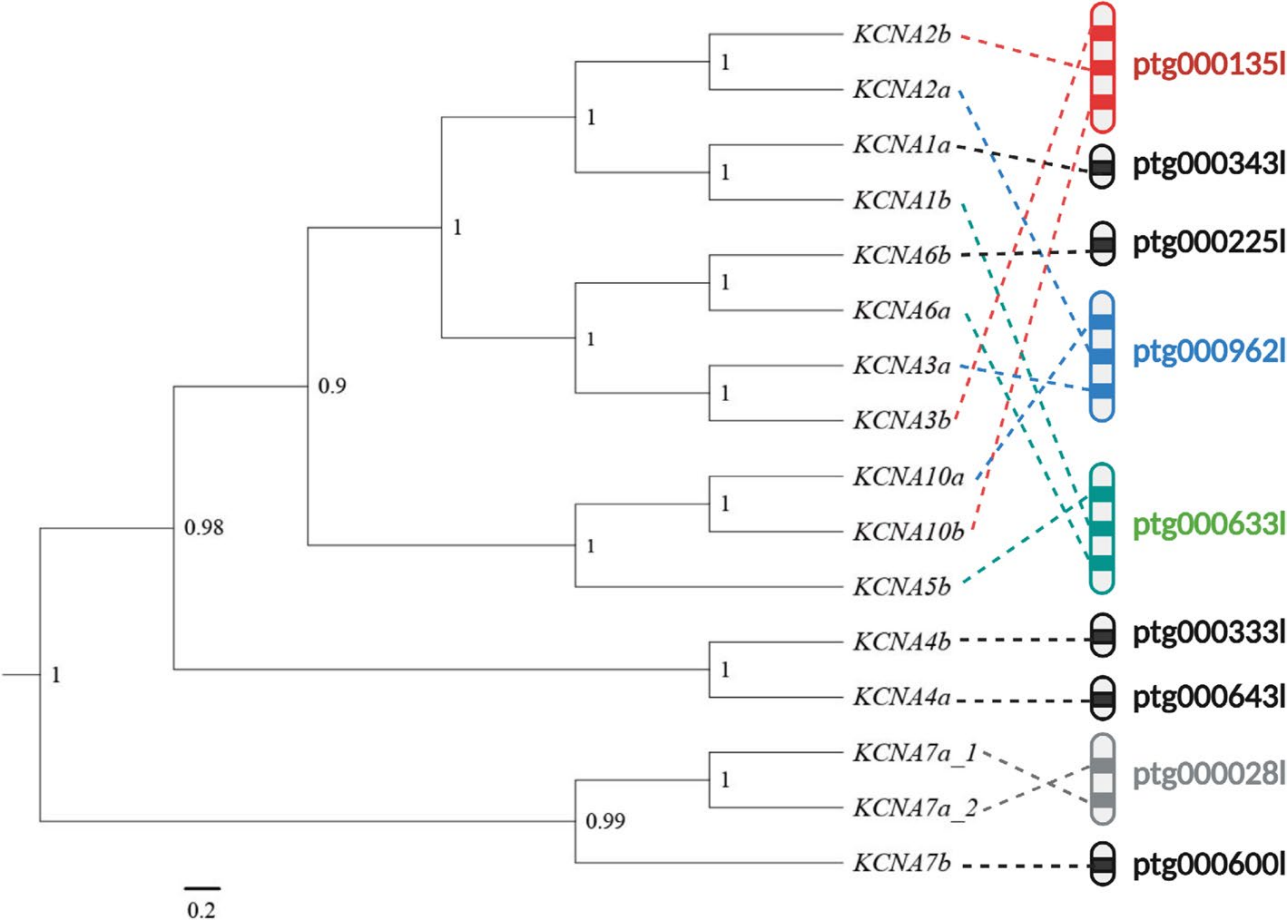
Bayesian tree of all curated *Kv1* genes in *C. compressirostris* genome. The posterior probability value is shown on each node. Genes are also mapped to their respective contig. The following genes are linked, i.e., mapped to the same contig: I: *KCNA2b, KCNA3b, KCNA10b*; II: *KCNA2a, KCNA3a, KCNA10a;* III: *KCNA1b, KCNA5b, KCNA6a;* IV: *KCNA7a_1, KCNA7a_2*

The phylogenetic analysis of all *Kv1* genes in BEAST v1.8.4 [38] suggests a basal position of *KCNA7a/b* genes (Fig. 6). The *KCNA1a/b* and *KCNA2a/b* form a monophyletic cluster, as do *KCNA5b* and *KCNA10a/b, KCNA3a/b* and *KCNA6a/b*. Those monophyletic gene clusters corroborate the hypothesis that the three clusters resulted from a complete duplication of the original cluster instead of independent tandem duplication [39].

## Discussion

### Genomic resources for electric fish and early teleost evolution

This is the fourth genome of an electric fish and the third well-annotated genome for the basal teleost taxon Osteoglossomorpha. Its quality is significantly improved with regard to contig length, when compared to the existing genomes for electric fish (*P. kingsleyae*, *E. electrophorus* [25, 26] and the non-electric Osteoglossomorpha fish *S. formosus* [40]. Regarding the number of contigs, completeness of BUSCO matches, and GC-content, our genome is comparable to the recently released *B. occidentalis* genome ([24]; a gymnotiform electric fish), which was generated by 10 × genomics linked read sequencing (Table 1). Our new genome will provide a valuable resource for future research on the evolution and ecology not only of electric fish, but also of the basal Osteoglossomorpha and early teleosts in general.

The draft annotation from MAKER predicted 34,492 protein-coding genes (Table 1). This is a substantially higher number than revealed in the annotations of *P. kingsleyae* (27,677) and *S. formosus* (22,016) [25] in the original publications. It had been hypothesized that mormyrid fishes may have a larger number of genes than non-electric osteoglossiformes [25], however, gene counts could also vary due to annotation artifacts, for example if reads from single genes are erroneously annotated to two genes [25], or uncollapsed haplotypes in the assembly. The annotations we used in our CAFE5 analysis were downloaded from NCBI pipeline, which resulted in 23,862 and 23,537 genes for *P. kingsleyae* and *S. formosus,* respectively, hence not supporting a generally increased gene number in mormyrids, relative to other Osteoglossomorpha fishes. To investigate whether the larger number of genes in *C. compresssirostris* is due to fragmentation of single genes in the annotation from MAKER, we mapped the CDS of *C. compressirostris* to the reference *P. kingsleyae* CDS from NCBI using MUMmer4.0 [41], allowing the *C. compressirostris* only map to the best hit in the reference (Additional file 5). This showed 3,076 overmapped genes in *C. compressirostris* that could indicate more than one gene model matching to a single gene model in *P. kingsleyae*. However, these could also be matches between similar genes and true duplications. In addition, there were an additional 10,846 and 2,939 unique genes in *C. compressirostris* and *P. kingsleyae*, respectively. These did not map between the species and suggests an excess of new and expanded genes in *C. compressirostris*, or lost/un-annotated genes in *P. kingsleyae*. This is also supported by the small subset of known genes that we looked at (i.e., the *Kv.1* genes), where we manually confirmed the genes are not overly predicted in the *C. compressirostris* genome annotation. It is possible that the larger gene number in *C. compressirostris* (relative to the other mormyrid *P. kingsleyae*) reflects true differences among these species. Our improved assembly with longer and fewer contigs may also have facilitated annotation of more genes.

### *KCNA* genes in *C. compressirostris* genome

It is generally accepted that there were two rounds of genome duplication in early vertebrate evolution and an additional genome duplication event in the ancestor of teleost fishes [39]. These ancient duplications could be reflected in the gene tree of the *Kv1* gene family. The monophyletic clusters of (1) *KCNA5b* and *KCNA10a/b*, (2) *KCNA1a/b* and *KCNA2a/b*, and (3) *KCNA3a/b* and *KCNA6a/b* are indeed compatible with a scenario of three subsequent whole genome duplications (Fig. 6). *KCNA7a/b* genes were also identified from our genome. These genes were the only ones containing an intron, while all other *KCNA* genes are intronless. Our manual curation showed that the *KCNA7a* gene has two gene copies, which are profoundly diverged in one of its exons. They are otherwise very similar and are situated close to each other in the same contig, pointing towards a recent lineage-specific tandem duplication. The *KCNA5a* was not found in the genome. This could reflect the incompleteness of our genome, but this copy could also have been lost during evolution [39]. Gene loss in this gene family is not uncommon among teleost fish, and in some lineages such as zebrafish, pufferfish and medaka only four monophyletic clusters in the *Kv1* gene family were found [39].

*Kv1* genes are hypothesized to be potentially involved in the diversification of the EOD signal among mormyrid weakly electric fish. 13 *Kv1* genes are upregulated in the EO in the species *C. tshokwe* (a species with an elongated EOD) compared with skeleton muscle and *C. compressirostris* (a species with short EOD; [19]). While the *KCNA7b* gene is considered to be upregulated in the skeleton muscle in mormyrids, the *KCNA7a* gene is predominantly expressed in the EO [42]. This points towards one of the duplicated gene copies having evolved a new function (neofunctionalization) [43]. This might have occurred in mormyrid fishes, leading to more diverse functions among *Kv1* genes. In particular, the evolution of an electric organ may have exerted different selection pressures on ion channels, such that one paralog may have evolved a new function (in the EO), while the other maintains the original state. This could have fostered the retention of many *KCNA* genes, in comparison to other non-electric teleosts. Here, it is particularly interesting that we found *Campylomormyrus* to possess an additional copy of the *KCNA7a* gene. Not only is this gene known to be predominantly expressed in the electric organ, but expressed sequence differences in this gene have also been discussed underlying length modulation of the EOD [42]. Indeed, EOD divergence is considered a major driver of the radiation within the genus *Campylomormyrus* [18]*.* We found the exon 2 of the two *KCNA7a* duplicates exhibiting numerous expressed sequence variations. This exon encodes for mediating the voltage-dependent potassium ion permeability of excitable membranes, and the possession of two putatively functional copies may hence have facilitated divergent EOD evolution in *Campylomormyrus*. This hypothesis, however, still awaits evaluation by functional studies.

### Gene family expansion and contraction in teleost

The teleost-specific whole genome duplication has shaped the evolutionary history of many teleost lineages by providing extensive raw materials for species radiation [6]. A likely fate of many duplicated genes is also that they can become non-functional [7] as a result of lacking the selective constraint on preserving both genes. This may explain the global pattern of more contracted than expanded gene families in most teleost species. This pattern is only reversed in the two species representing Salmonidae and Cyprinidae, both having experienced an additional WGD [44, 45].

According to our CAFE5 analysis, Osteoglossomorpha appear to have a more rapid gene family turnover rate (lambda) than Otomorpha and Euteleosteomorpha (Fig. 2 & 3). In particular, we found a significantly higher expansion/contraction ratio in Osteoglossomorpha, relative to other teleost lineages. This is exemplary supported by the *Kv1* gene family. Eight *Kv1* gene clusters (in total of 16 genes) were curated in *C. compressirostris* and an additional gene duplication detected (duplicating *KCNA7a*), while in other species such as the pufferfish, medaka, stickleback and zebrafish there are only four clusters. Although this is only a single gene family, it suggests a possible scenario of subfunctionalization and neofunctionalization in particular in the lineages with an active electric sense, which may contribute to the higher turnover rate and expansion/contraction ratio in Osteoglossomorpha.

### Pathway evolution in Mormyroidea

Pathway enrichment analysis is a tool to infer biologically relevant genes and biological processes from high-throughput data. The pathway of primary bile acid biosynthesis was most prone to gene family contraction in African weakly electric fish (mormyrids). This pathway takes place in the liver of vertebrates [46], where the synthesized bile acid can be conjugated with taurine or glycine before secretion via bile into the intestine. The pathway with most gene family expansion among mormyrids is nitrogen metabolism, one of the pathways for forming nitrogenous endproducts from protein degradation [47]. The expanded gene families contained within this second pathway are mostly related to carbonic anhydrases (e.g. CA12, CA4). These genes help maintaining acid–base homeostasis, regulating PH, and perhaps most relevantly, they play an active role in ion uptake [48]. It has been shown in mammals that genes such as carbonic anhydrases CA2 and CA4 play important roles in epithelial acid secretion and sodium uptake [48]. Although we do not know the expression pattern of those CA genes in mormyrids, they might be involved in ion transport as well, especially of potassium and sodium, which are key to generate electric signals.

Expanded specifically in the electric mormyrids were gene families of the Wnt signaling pathway, encoding for a wide array of cellular processes including cell fate determination, motility, polarity, primary axis formation and organogenesis. It can be divided into the Planar Cell Polarity pathway and the Wnt/Ca^2+^ pathway. High turnover was also observed in the calcium signaling pathway, which is mostly contracted in both mormyrid species and their common ancestor. It regulates the Ca^2+^ entering the cell from the outside. It was found to be down-regulated in the EO compared with skeleton muscle [21], which may have resulted from the contracted OGs of this pathway.

## Conclusions

A new high-quality genome of an African weakly electric fish (*C. compressirostris*, Mormyridae) is reported here, representing an important contribution to understand the evolution of electric fish and Osteoglossomorpha fish genomes. Our gene family analysis relative to representatives of many teleost fish genomes reveals a more rapid turnover rate and a higher expanded/contracted gene family number ratio in Osteoglossomorpha. The functional importance of these gene families requires further investigation, but provides many avenues for understanding the unique adaptations in these fishes. We also identified most of the *KCNA* gene clusters in our genome except for *KCNA5a*. The *KCNA7a* gene was found to be tandem duplicated. *KCNA* genes are considered of prime importance in the evolution of the active electric sense in teleosts. Our exhaustive efforts to localize these genes (including detection of a novel tandem duplication) underline the potential our new genome may hold towards an improved understanding of electric fish and Osteoglossomorpha evolution.

## Methods

### Samples

Genomic DNA was isolated from available frozen fin clips, which had been previously taken in the course of another study from an adult *C. compressirostris* artificially bred and raised at University Potsdam, Germany. The CTAB protocol was used to obtain high molecular weight genomic DNA [49]. The concentration and quality were further verified with Nanodrop spectrophotometer and Agilent TapeStation before sequencing.

### Genome sequencing

For Pacbio sequencing, a 15-kb SMRT cell DNA library was prepared and sequenced on a PacBio Sequel platform with one SMRT cell by a commercial company (Novogene). This produced 294 Gb long reads, which were used to generate the HiFi long reads using circular consensus sequencing (CCS) mode (Pacific Biosciences, USA).

### De novo genome assembly

The genome size and heterozygosity was estimated by GenomeScope 2.0 [50] using a k-mer value of 32 [28]. The genome was further assembled by hifiasm [29] with the HiFi reads as input. The separated primary haplotigs were visualized in Bandage [51]. This showed that some of the contigs contain two forks, which are likely homozygous breakpoints. Therefore, the program purge_dups [52] was additionally applied for haplotig purging in the primary haplotigs. The mitochondrial DNA was separately assembled with the MitoHiFi.

We examined potential contamination using Blobtools2 [53] based on divergence in GC-content and coverage. We further assessed the presence of core, single copy and orthologous genes through BUSCO 5.3 [30] with the actinopterygii_odb10 orthologues as reference.

### Genome annotation

Before annotation, we performed repeat masking in RepeatModeler 1.0.11 [31] provided in GenSAS v6.0 [54]. The soft repeat-masked sequence was used as an input in MAKER [55]. To provide EST evidence, we assembled the transcript sequences from Lamanna et al*.* [21] and newly generated RNA sequence data (Feng & Tiedemann, unpubl. results) of *C. compressirostris* with Trinity [56]. 575,330 transcripts were assembled by Trinity from electric organ and skeleton muscle tissues, and they were all used as EST evidence in MAKER. In addition, 244,298 protein sequences were collected from all vertebrate proteins in NCBI.

The soft-masked assembly was predicted in MAKER with different gene predictors in three steps. In the first round, the RNA and protein sequences were supplied as evidence, and trained with the ab initio gene predictors SNAP [57] and Augustus [58] based on BUSCO. In the second round, we created a new SNAP-HMM input file based on the first round output and repeated the run with the same parameters as in the first round. The output from the second run was further analyzed in the third round following the method in the second round. The final output of the predicted gene, exon and intron information was statistically summarized by GAG [59]. We also manually checked the *KCNA* genes (see below) to exemplary confirm annotation quality. All the CDS were used to identify conserved protein domains with InterProScan [60].

### Gene family expansion and contraction analysis

In order to get an insight to the evolutionary dynamics of the genome evolution, the gene family analyzer CAFE5 [32] was used to infer expansion and contraction of gene ortholog clusters in 33 teleost fish. The species were selected such that they represent the taxonomic diversity among teleosts and include the only three available species from Osteoglossomorpha, six species from Otomorpha and 24 species from Euteleosteomorpha. A further selection criterion was genome quality, i.e. all selected representative species have a genome contig N50 over 100 kb, except for that of *P. kingsleyae*, which was though retained, as it comprises the only other genome from an African electric fish (Mormyridae). We obtained the peptide sequences from those genomes in NCBI and retained the longest isoform for each peptide. Gene families (orthogroups) were clustered among the filtered peptide sequences from all selected species in OrthoFinder [33], using an all-vs-all BLAST [38] for sequence similarity searches. Gene gain and loss in each lineage were calculated in CAFE5 with a random birth–death process model, based on the ultrametric species tree, which was generated by OrthoFinder using Fasttree [61]. Taxon-specific lambda values (rates of evolutionary change) were estimated for Osteoglossomorpha, Otomorpha and Euteleosteomorpha.

In order to compare the gene family expansions and contractions relative to the most recent common ancestor (MRCA) of all selected teleost fishes, we summarized the gene and gene family change for each species and counted the ratios of expanded/contracted gene family (and gained/lost gene) numbers. A t-test was used to compare the ratios of expanded/contracted gene family numbers between Osteoglossomorpha & Euteleosteomorpha and Osteoglossomorpha & Otomorpha. Note that we only performed these two pairwise comparisons. The testing scheme is hence orthogonal and does not require any further correction [62].

We calculated the total number of orthogroups (OGs) from OrthoFinder and the number of significantly expanded and contracted OGs (*P*-value less than 0.05) among all species from the CAFE5 analysis. These OGs were separately blasted against the UniProt database. We further counted the OG number that showed up the most (zinc finger, transposon, immunoglobulin and GTPase) in both datasets, i.e. among all the OGs and the significantly contracted/expanded OGs. A Fisher’s exact test [34] was then applied to identify significant deviations in the number of contracted/expanded OGs of each functional category, relative to the numbers of contracted/expanded OGs among all annotated OGs.

We collected the OGs inferred from electric fish and Osteoglossomorpha genomes (*C. compressirostris, P. kingsleyae, E. electricus* and *S. formosus*) and used the program VennDiagram [37] to visualize shared/unique OGs number among those four species. For *C. compressirostris, P. kingsleyae* and the ancestor node (as representative of Mormyridae), we performed an enrichment analysis by assigning contracted and expanded OGs to metabolic pathways using the KEGG database [36].

### *KCNA* gene clusters curation

In total, we collected 233 *KCNA* genes sequences of teleost fishes in NCBI and blasted them against our genome. We identified each *KCNA* genes based on an e-value less than 1e-6 and the best raw score from blast output. The identified *KCNA* genes were reciprocally blasted in NCBI. After we curated all found *KCNA* genes in our new *C. compressirostris* genome, a phylogenetic Bayesian tree was built in BEAST v1.8.4 [63] using a GTR + G substitution model, a relaxed lognormal clock model, the Yule speciation model and one billion MCMC. The result was preserved only if the effective sample size (ESS) were all over 200. 10% of the starting MCMC were used as burn in and the remainder was used to generate a phylogenetic tree.

## Supplementary Information

**Additional file 1.** Annotation edit distance (AED) score distributions for the *C. compressirostris* annotation by MAKER.

**Additional file 2.** Information on teleost genomes used in the CAFE5 analysis.

**Additional file 3.** Number of gene and gene family expansions and contractions compared with MRCA, and ratio of the expanded/contracted gene family (gain/loss gene) numbers.

**Additional file 4.** KCNA gene sequences.

**Additional file 5.** Mummer alignment between the CDS of *C. compressirostris* and *P. kingsleyae*.

## Data Availability

The genome datasets generated during the current study are available in the European Nucleotide Archive under the accession number GCA_910591475 at https://www.ebi.ac.uk/ena/browser/view/GCA_910591475.1. The raw sequencing reads, assembled genome, annotation as well as the *KCNA* genes sequences were stored in Dryad under the DOI: https://doi.org/10.5061/dryad.c59zw3rcj.
