# Supplementary material for: A new genome assembly of an African weakly electric fish (Campylomormyrus compressirostris, Mormyridae) indicates rapid gene family evolution in Osteoglossomorpha
Source: BMC Genomics. 2023 Mar 20;24:129. doi: 10.1186/s12864-023-09196-6 (PMC10029256; doi:10.1186/s12864-023-09196-6)
Supplement: Supplementary file 1 — Additional file 1. Annotation edit distance (AED) score distributions for the C. compressirostris annotation by MAKER. [file 12864_2023_9196_MOESM1_ESM.pdf]

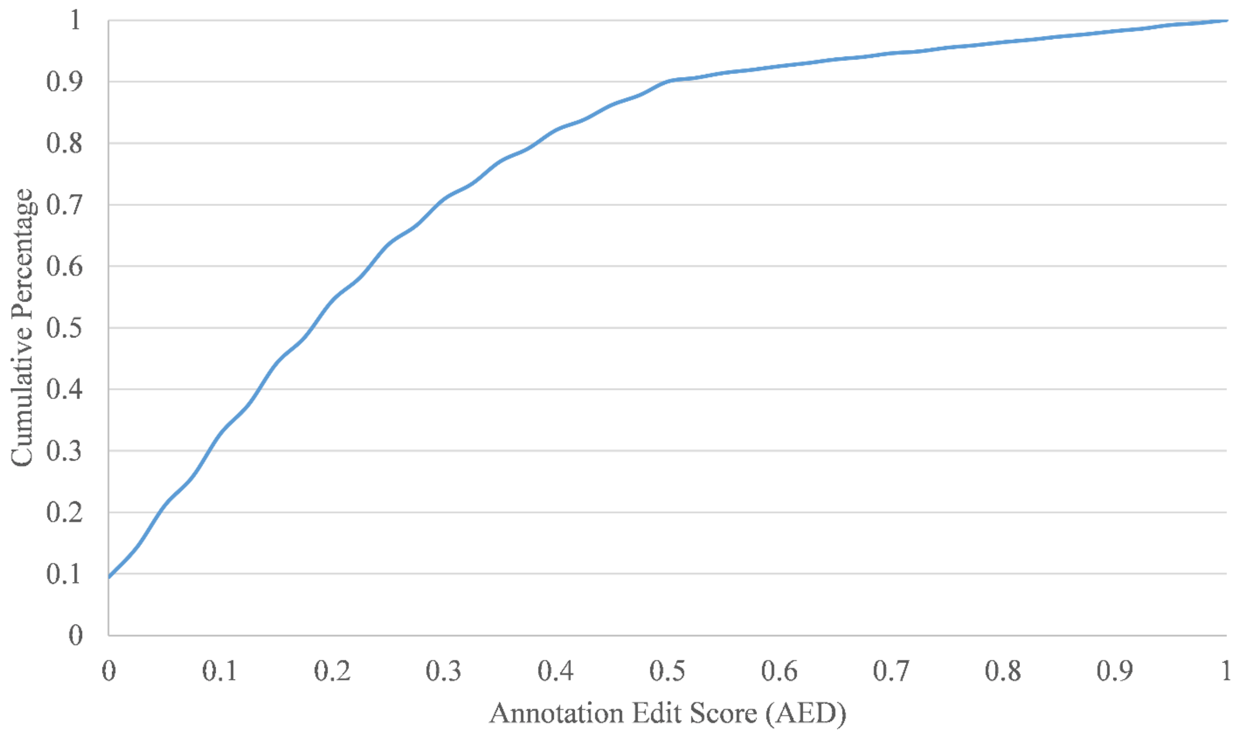

Additional file 1: Annotation edit distance (AED) score distributions for the *C. compressirostris* annotation by MAKER
