# Supplementary figures and images for: A new genome assembly of an African weakly electric fish (Campylomormyrus compressirostris, Mormyridae) indicates rapid gene family evolution in Osteoglossomorpha
Source: BMC Genomics. 2023 Mar 20;24:129. doi: 10.1186/s12864-023-09196-6 (PMC10029256; doi:10.1186/s12864-023-09196-6)

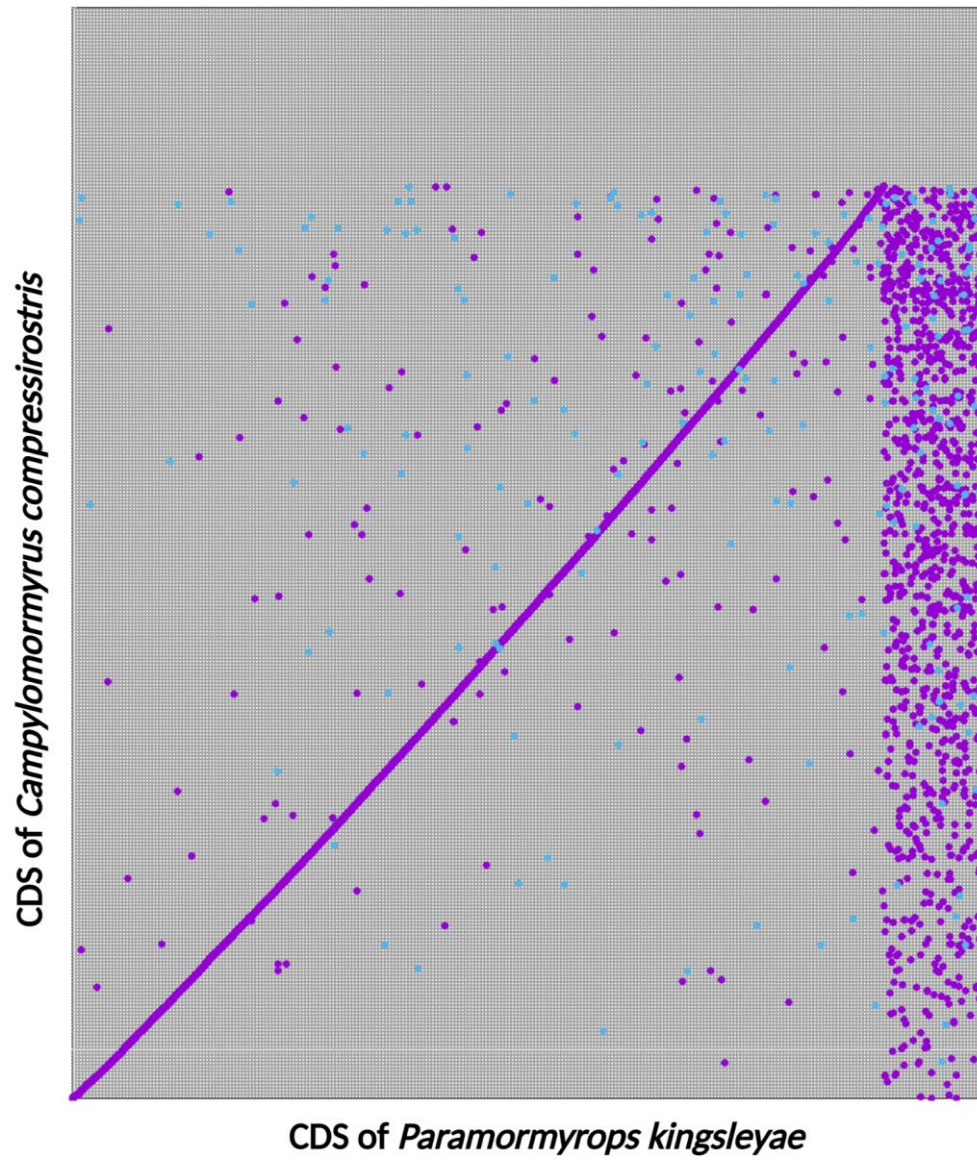

**Additional file 5:** Mummer alignment between the CDS of *C. compressirostris* and *P. kingsleyae*.

Supplement: Supplementary file 5 — Additional file 5. Mummer alignment between the CDS of C. compressirostris and P. kingsleyae. [file 12864_2023_9196_MOESM5_ESM.pdf]
